
JOURNAL INFORMATION
==============================
NLM Title Abbreviation: ScientificWorldJournal
Journal ID: ScientificWorldJournal
NLM Journal ID: 101131163
Journal ID: TSWJ

The Scientific World Journal

ISSN: 2356-6140
EISSN: 1537-744X
Publisher: Wiley

ARTICLE INFORMATION
==============================
PMCID: PMC6084222
PMID: 30147601
DOI: 10.1100/tsw.2001.218
Article ID: 638969
Article version: 1
Subjects: Short Report

Macrophage Apoptosis Induced by an Avian Strain of Escherichia coli

Horn Fabiana *
Bastiani Michele
Santos Diógenes S.
Departamento de Biofísica and Departamento de Biotecnologia, Universidade Federal do Rio Grande do Sul, PO Box 15005, Porto Alegre 91501-970, Brazil
*Fabiana Horn: fhorn@dna.cbiot.ufrgs.br
Publication date: 2001
Electronic publication date: 2001 May 1
Volume: 1
First page: 120
Last page: 120
Copyright: Copyright © 2001 Fabiana Horn et al.
Copyright year: 2001
License: This is an open access article distributed under the Creative Commons Attribution License, which permits unrestricted use, distribution, and reproduction in any medium, provided the original work is properly cited.
License URL: https://creativecommons.org/licenses/by/3.0/

Funding: Fundação de Amparo à Pesquisa do Estado do Rio Grande do Sul

==============================
Miami *Nature Biotechnology* Short Reports
*TheScientificWorld* (2001) 1 (S3), 120SR
ISSN 1532-2246; DOI 10.1100/tsw.2001.218

### MACROPHAGE APOPTOSIS INDUCED BY AN AVIAN STRAIN OF *Escherichia coli*

Fabiana Horn*, Michele Bastiani, and Diógenes S. Santos

Departamento de Biofísica and Departamento de Biotecnologia, Universidade Federal do Rio Grande do Sul, PO Box 15005, Porto Alegre 91501-970, Brasil
* fhorn@dna.cbiot.ufrgs.br

**INTRODUCTION.** The avian *Escherichia coli* strain UEL17 has been reported to cause apoptosis in mouse peritoneal phagocytes (1), as judged by TUNEL assay, electrophoresis of total DNA and HE staining. Here we show that UEL17 causes cell death also in the macrophage cell line J774, as observed by LDH liberation, and tested the involvement of 5 caspases in this process.

**METHOD**. Macrophages J774, plated at $7{,}5 \times 10^4$ cell/cm$^2$, were infected with UEL17 or M90T (positive control, ref. 2) in RPMI 1640 plus glutamine. After 1 h, cells were washed and incubated further in the presence of gentamicyn 50 µg/ml to complete 4 h from infection. LDH was measured using the Cytotox96 kit (Promega), following the manufacturer´s instructions. To measure caspase activity, cells were lysed and the extracts incubated with 5 fluorogenic substrates; the reaction was monitored at 370/460 nm.

**RESULTS.** J774 cells infected with UEL17 at a multiplicity of infection (MOI) of 20, 50 and 150 bacteria per cell presented a corresponding number of internalized bacteria (below, a, b and c); when M90T was used (MOI of 150), J774 cells showed an average of 5 bacteria per cell. The LDH liberation (Fig. 1; control ■; M90T ○; UEL17 ●) from cells infected with either bacteria (both at a MOI of 150) indicated cell death, in agreement with previous results (1, 2). For caspase activation, we tested the hydrolysis, by the cell extracts, of the following substrates: Ac-YVAD-MCA (for caspase1/4), Ac-DEVD-MCA (caspase 3/7), Ac-VEID-MCA (caspase-6), Ac-IETD-MCA (caspase 6/8), Ac-LEHD-MCA (caspase-9). We only detected hydrolysis of the substrate

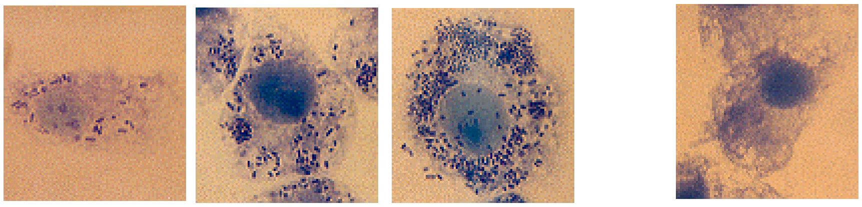

a b c control

**Fig. 1** **Fig. 2**

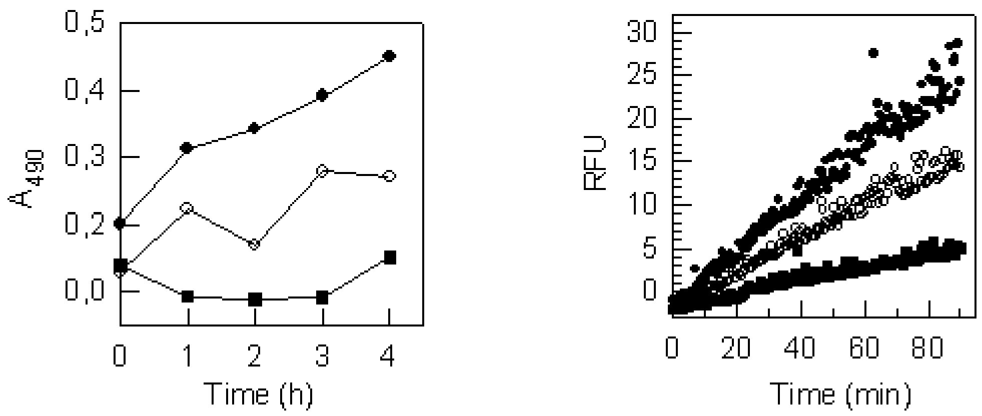

Ac-DEVD-MCA (Fig. 2; control ■; M90T ○; UEL17 ●), indicating that caspase-3/7 is involved in apoptosis induced by UEL17 infection.

**DISCUSSION.** Macrophage apoptosis caused by UEL17 infection involves caspase-3 activation. Since caspase-8 does not appear to be activated, as expected if apoptosis was triggered by Fas activation, it is possible that UEL17 interferes directly with a caspase downstream of caspase-8.

**ACKNOWLEDGEMENTS.** This work was supported by Fundação de Amparo à Pesquisa do Estado do Rio Grande do Sul (FAPERGS).

**REFERENCES.**

1. Rodrigues, V.S., Vidotto, M.C., Felipe, I., Santos, D.S., and Gaziri, L.C.J. (1999) FEMS Microbiol Lett. 179, 73
2. Zychlinsky, A., Prevost, M.C., and Sansonetti, P.J. (1992) Nature 358, 167
